# Reducing contrast media dosage for pulmonary embolism CTPA in PCD-CT: a comparative study of EID-CT and PCD-CT in the era of individualized protocolling

**DOI:** 10.1007/s00330-025-12054-6

**Published:** 2025-10-17

**Authors:** Lion Stammen, Eva J. I. Hoeijmakers, Thomas G. Flohr, Hester A. Gietema, Janneke Vandewall, Joachim E. Wildberger, Cécile R. L. P. N. Jeukens, Bibi Martens

**Affiliations:** 1 Department of Radiology and Nuclear medicine, Maastricht University Medical Centre+, Maastricht, The Netherlands; 2 CARIM Cardiovascular Research Institute Maastricht, Maastricht University, Maastricht, The Netherlands; 3 Siemens Healthineers, Forchheim, Germany; 4 GROW Research Institute for Oncology and Reproduction, Maastricht University, Maastricht, The Netherlands

**Keywords:** Computed tomography, Diagnostic imaging, Pulmonary artery, Contrast media, Photon-counting detector

## Abstract

**Objectives:**

To evaluate diagnostic image quality (IQ) for pulmonary embolism detection in photon-counting detector CT (PCD-CT) with significantly reduced contrast media (CM) dose vs conventional energy-integrating detector CT (EID-CT), using optimized and individualized CM protocols.

**Materials and methods:**

Consecutive CT pulmonary angiography (CTPA) scans performed on the EID-CT (Jan 2024–Mar 2024) with an individualized kilovoltage (kV) and total body weight (TBW) adapted CM protocol, and on the PCD-CT (Aug 2023–Feb 2024) with a TBW adapted CM protocol matching the 70 kV EID-CT protocol, were retrospectively collected. EID-CT scans were performed at 70–120 kV, based on patients’ size, while PCD-CT scans were performed at 120 kV with 55 keV virtual monoenergetic images. Objective IQ assessment included mean attenuation (in Hounsfield Units), signal-to-noise ratio, and contrast-to-noise ratio (CNR). Two board-certified radiologists assessed diagnostic IQ subjectively using a five-point Likert scale.

**Results:**

In 140 EID-CT and 118 PCD-CT scans, PCD-CT reduced total iodine load by 26.7% and CT dose index volume by 24.4%. Objective IQ parameters showed no significant differences, except for a decrease in CNR in the proximal pulmonary arteries in PCD-CT scans (*p* = 0.02). Subjective IQ was rated as moderate/good by observers 1 and 2 in 94.9% and 97.9% of the EID-CT scans, respectively, and 96.5% and 100% of the PCD-CT scans, respectively.

**Conclusion:**

PCD-CT with a TBW-adapted CM protocol can achieve substantial reductions in both CM and radiation dose compared to EID-CT with individualized kV and TBW adapted CM protocols, while maintaining diagnostic IQ in CTPA scans.

**Key Points:**

***Question*** *The feasibility of CM reduction in PCD-CT vs EID-CT with highly individualized CM protocols, while maintaining diagnostic IQ*.

***Findings*** *PCD-CT reduces total iodine load by 26.7% and CT dose index volume by 24.4% compared to EID-CT, with comparable objective and subjective diagnostic IQ*.

***Clinical relevance*** *This approach enables CM reduction, potentially lowering patient risk and providing environmental and financial benefits. Additionally, PCD-CT allows for CM and radiation dose reduction while maintaining comparable IQ*.

**Graphical Abstract:**

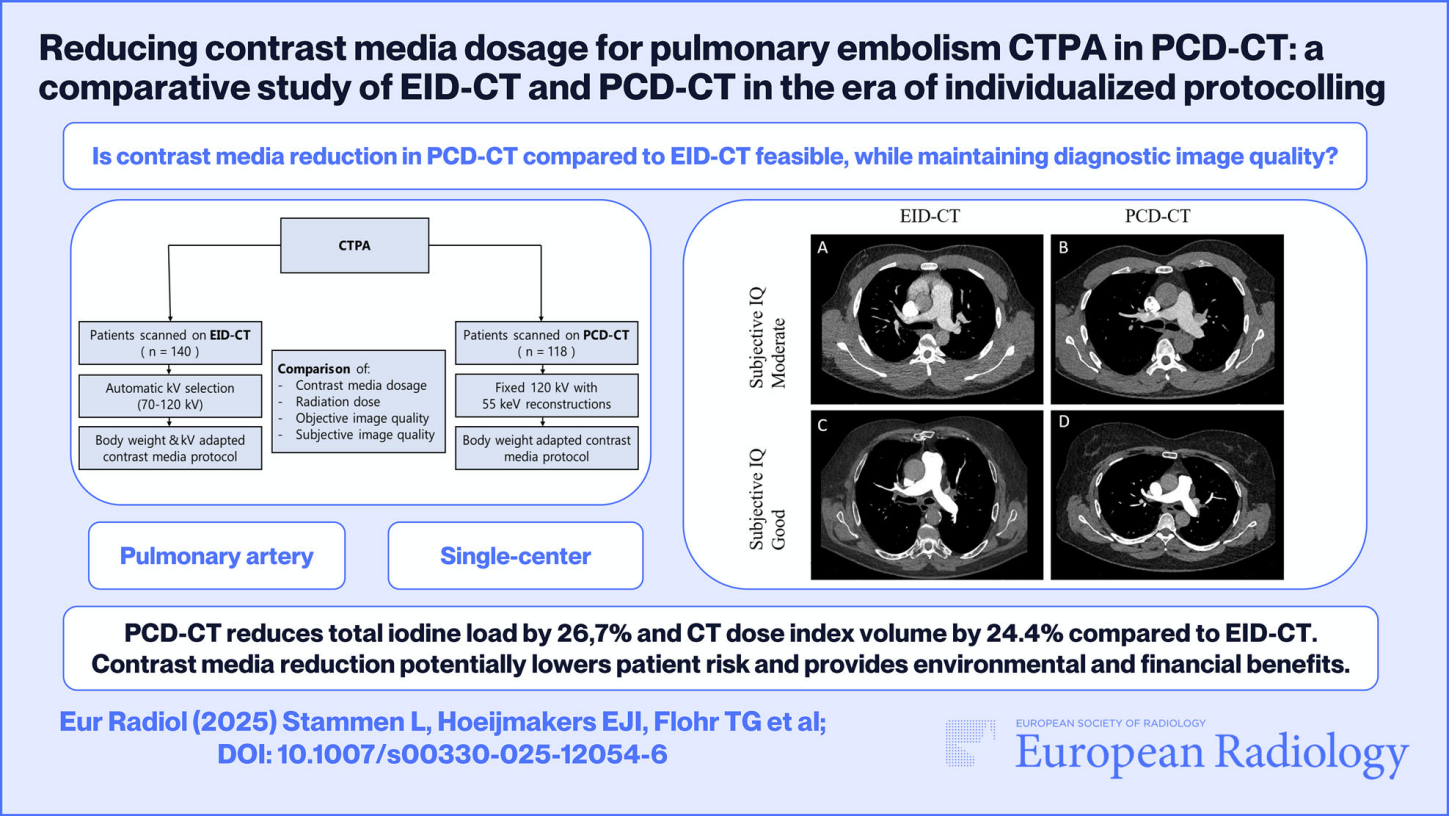

## Introduction

Computed tomography pulmonary angiography (CTPA) has become the cornerstone for diagnosing pulmonary embolism (PE), providing high-resolution imaging of the pulmonary vasculature [1–3]. Previous studies demonstrated a high sensitivity (96–100%) and specificity (89–98%), establishing CTPA as the gold standard technique for diagnosing PE [4]. The use of iodinated contrast media (CM) is integral to CTPA imaging, facilitating the visualization of pulmonary arteries and the identification of emboli as areas without CM. However, the administration of CM is not without concerns or side effects, including potential life-threatening contrast-induced acute kidney injury (CI-AKI) in patients with renal insufficiency. Though the severity of the incidence and risks associated with CI-AKI is still a subject of ongoing debate, caution is recommended in patients with severe pre-existing renal insufficiency [5–10]. Additionally, excess CM can end up in wastewater, so minimizing its use can help reduce the ecological footprint of medical practices [11, 12]. This approach also contributes to addressing the ongoing CM shortage and offers financial benefits [13].

Previous studies focused on optimizing CM protocols for CTPA to reduce CM dose while preserving diagnostic image quality (IQ) [14–17]. A previous study has demonstrated that total body weight (TBW) adapted CM protocols lead to more homogenous attenuation and reduced CM dose for CTPA imaging [17]. Furthermore, adapting the CM dose to the optimal kilovoltage (kV) setting, following the simple 10-to-10 rule (reduction of the CM dose by 10% when the kV setting is reduced by 10 kV) facilitates the implementation of highly individualized CM protocols, tailored to both tube voltage and TBW [18, 19].

In recent years, advancements in CT technology have resulted in the introduction of photon-counting detector CTs (PCD-CTs) [20–22]. As opposed to an energy-integrating detector CT (EID-CT), PCD-CT enables the counting of individual X-ray photons, which allows detection of their energies and sorting them in several energy bins. Lower energy photons contribute more strongly to the image than in EID-CT, leading to increased image contrast particularly in contrast-enhanced CT scans. In addition, the electronic noise is eliminated, as it is below the threshold energy for counting [23]. Therefore, image noise is reduced with PCD-CTs [22], and in combination with increased image contrast, this leads to an inherently higher iodine contrast-to-noise ratio (CNR). The available spectral data provided when using standard scan mode enables reconstruction of virtual monoenergetic images (VMI) at various energy (keV) levels. For CTPA, the ability to reconstruct VMIs at lower keV levels, along with higher CNR, provides the opportunity to adapt CM and scan parameters while maintaining sufficient IQ [24].

Previous studies suggest that reduced CM dose or reduced radiation dose using PCD-CT still results in sufficient IQ in CTPA [25–28], in CT angiography [29, 30], and in abdominal CT [31, 32]. However, for CTPA, the CM reduction potential of PCD-CT with TBW-adapted CM protocols, compared to highly individualized, kV- and TBW-adapted CM protocols in EID-CT, has not yet been evaluated.

This study aims to investigate the feasibility of reducing CM doses while maintaining diagnostic IQ for PE detection with PCD-CT compared to conventional EID-CT, using optimized routine protocols for both scanners. In EID-CT, the kV is automatically adapted to the patient’s morphology, and the CM protocol is adapted to both kV and TBW. For PCD-CT, a standardized scan protocol at 120 kV with standardized reconstruction of VMIs at 55 keV is used for all patients, and the CM protocol is only adapted to TBW.

## Material and methods

### Ethics

This study received a waiver of written informed consent from the local ethical committee and institutional review board (METC 2020-1535-A-1), due to its retrospective nature.

### Study population

Consecutive CTPA scans used in daily clinical practice were collected retrospectively from a third-generation dual-source EID-CT system (SOMATOM Force; Siemens Healthineers, between Jan 2024–Mar 2024) and a first-generation dual-source PCD-CT (NAEOTOM Alpha; Siemens, between Aug 2023–Feb 2024). Scans of patients ≥ 18 years were included that were solely conducted to rule out PE. Scans with adjustments to the CM protocol for diagnosis of other potential pathologies, such as aortic dissection or coronary assessment, were excluded from the analysis. Additionally, scans with obvious breathing artifacts were excluded, as were scans with modified CM dose or kV settings (PCD-CT). The picture archiving and communication system (PACS) workstation (IDS7 version 24.2; Sectra AB) and the CM data acquisition program (Certegra Informatics Solution; Bayer Healthcare) were consulted to collect patient characteristics (age, gender, weight, height).

### Scan protocol

The EID-CT scans were conducted with a gantry rotation time of 0.25 s and a pitch of 1.3 using single-energy (SE) mode. Other scanner settings were: slice collimation of 192 × 0.6 mm, a quality reference tube current of 105 mAs, and a reference tube voltage of 100 kV_ref_, with automatic tube current and tube voltage adaptation (CARE Dose 4D and CARE kV, Siemens), resulting in tube voltages ranging from 70 kV to 120 kV with increments of 10 kV. PCD-CT scans had a gantry rotation time of 0.25 s, a pitch of 2.4, and a slice collimation of 144 × 0.4 mm. The scans were made in the QuantumPlus mode, providing spectral information in four energy bins, which are combined into two bins for all established dual-energy (DE) applications. In PCD-CT, a fixed tube voltage is utilized to provide the full spectral data. For this study, a tube voltage of 120 kV was applied, with reconstruction of VMI’s at low keV as a substitute for low kV scanning. The reference IQ level (CARE keV IQ level) was set at 70 with automatic adaptation of the tube current and the VMI keV-level (CARE Dose 4D and CARE keV, Siemens), optimized for vascular studies. Reconstructions consisted of 1.0 mm slices with 0.7 mm (EID-CT) and 0.8 mm (PCD-CT) increments, using a soft tissue kernel (Bv40) and (quantum) iterative reconstruction strength 3. The PCD-CT scans were reconstructed using a VMI level at 55 keV as proposed by CARE keV. Radiation dose information, including CT dose index volume (CTDI_vol_), in mGy and tube voltage, was obtained from the digital imaging and communications in medicine (DICOM) header.

### CM injection protocol

All patients were injected with iodinated CM (Ultravist 300 mgI/mL or Ultravist 370 mgI/mL; Iopromide, Bayer) at room temperature, followed by a saline chaser (40cc) [33]. A programmable CM power injector was used for CM injections (Centargo; Bayer) [34]. The CM was administered with a fixed injection duration of 8 s, regulated by dedicated CM injection software (P3T; Bayer). Prior to the CTPA, a dynamic test bolus with an injection duration of 2–3 s, followed by a saline chaser (40cc), was obtained to determine the optimal scan delay (time to peak enhancement in the pulmonary trunk + 7 s), accounting for patient-specific factors like cardiac output [35, 36]. On the EID-CT scanner, a CM injection protocol individualized to tube voltage and TBW was used, according to the 10-to-10 rule, as described elsewhere [18, 19]. The TBW adjustment followed a non-linear relationship, as detailed and visualized by Hendriks et al [18]. For example, at 120 kV, an iodine dose of 0.257 g I/kg was used for a 70 kg patient, the dose was adapted to 0.240 g I/kg for a 90 kg patient, and 0.215 g I/kg for a 110 kg patient. Injection duration is kept constant at approximately 7 s, therefore influencing the IDR. On the PCD-CT, a VMI level of 55 keV was used, which is an estimate of the mean energy of a 70 kV spectrum. While this is only an approximation and does not imply the equivalence of 70 kV images and 55 keV VMIs, it provides a reasonable assumption for determining the CM protocol for PCD-CT. Therefore, a CM protocol identical to the 70 kV CM protocol used for the EID-CT scanner was used [33]. CM protocols were defined by a dosing factor (DF, gI/kg) that prescribes the amount of iodine in grams per kilogram. This ensures that the total iodine load (TIL, gI) was independent of the CM concentration used (300 mgI/mL or 370 mgI/mL).

A dedicated data acquisition program (Certegra Informatics Solution; Bayer) recorded the CM parameters for each patient, including the iodine concentration (g/mL), the total CM dose (mL), and the flow rate (mL/s). The TIL, iodine delivery rate (IDR), and DF were calculated as follows: TIL = [total CM dose × iodine concentration] in grams iodine (gI); IDR = [flow rate × iodine concentration] in grams iodine per second (gI/s); DF = [TIL/TBW] in grams iodine per kg body weight (gI/kg).

### IQ assessment

Objective assessment of IQ included the quantification of the mean attenuation (in Hounsfield Units, HU) as the primary outcome, and signal-to-noise ratio (SNR), and CNR as secondary outcomes. Regions of interest (ROIs), maximized in size, were drawn to measure the mean attenuation in HU and the standard deviation (SD). ROIs were placed in four specific anatomical locations: proximal in the left and right pulmonary artery, and subsegmental in a branch of the upper left pulmonary artery and of the lower right pulmonary artery. If not possible, for example due to the presence of PE or post-operative status, an adjacent vessel at the same side was chosen (e.g., if the upper left could not be used, the lower left pulmonary artery was chosen, and vice versa for the right side). In addition, an ROI was placed in the left paraspinal muscle. The proximal SNR was calculated by dividing the mean attenuation of left and right proximal pulmonary arteries by the mean SD. The distal SNR was calculated similarly, by dividing the mean attenuation of the distal pulmonary arteries by its mean SD. The CNR for the proximal pulmonary arteries was calculated by subtracting the attenuation of the paraspinal muscle from the mean attenuation of the proximal pulmonary arteries and dividing this difference by the SD of the paraspinal muscle. The distal CNR for the subsegmental pulmonary arteries was calculated accordingly.

Subjective IQ was assessed independently by two board-certified radiologists, H.G. and B.M., with respectively 18 years’ and 8 years’ experience in thoracic and cardiovascular imaging. Prior to the evaluation, the radiologists jointly reviewed ten additional scans (5 performed on the EID-CT and 5 on the PCD-CT), not included in the dataset, to familiarize themselves with the areas of focus and to systematically align for the subjective assessments. Scans were reviewed under clinical viewing conditions on diagnostic screens with standard window level settings for CTPA scans (with the flexibility to adjust settings). Both radiologists were completely blinded to all scans, reconstruction, and patient information. Scans were rated for diagnostic confidence (confidence to accurately diagnose presence or absence of PEs) using a 5-point Likert scale (1 = very poor, 2 = poor, 3 = moderate, 4 = high, 5 = excellent) in which a Likert score of 3 or higher indicated sufficient diagnostic confidence. For the analysis, Likert scores 1 and 2, and 4 and 5 were grouped as poor and good IQ, respectively.

### Statistical analysis

Statistical analysis was performed using SPSS statistical software package version 28 (IBM Corp). Continuous data were reported as mean ± SD, and a *p*-value < 0.05 was considered statistically significant. The normality of continuous data was assessed using the Shapiro–Wilk test. If data followed a normal distribution, an independent sample *T*-test was conducted; if not, the Mann–Whitney *U*-test was used. Categorical variables were expressed as percentages, and proportions were evaluated using a Chi-squared test.

In order to evaluate interobserver agreement in subjective IQ assessment between the two observers, the intraclass correlation coefficient (ICC) was calculated. Moreover, the percentage of scans that were rated as moderate or good was assessed, reflecting sufficient diagnostic confidence.

## Results

**Fig. 1:**
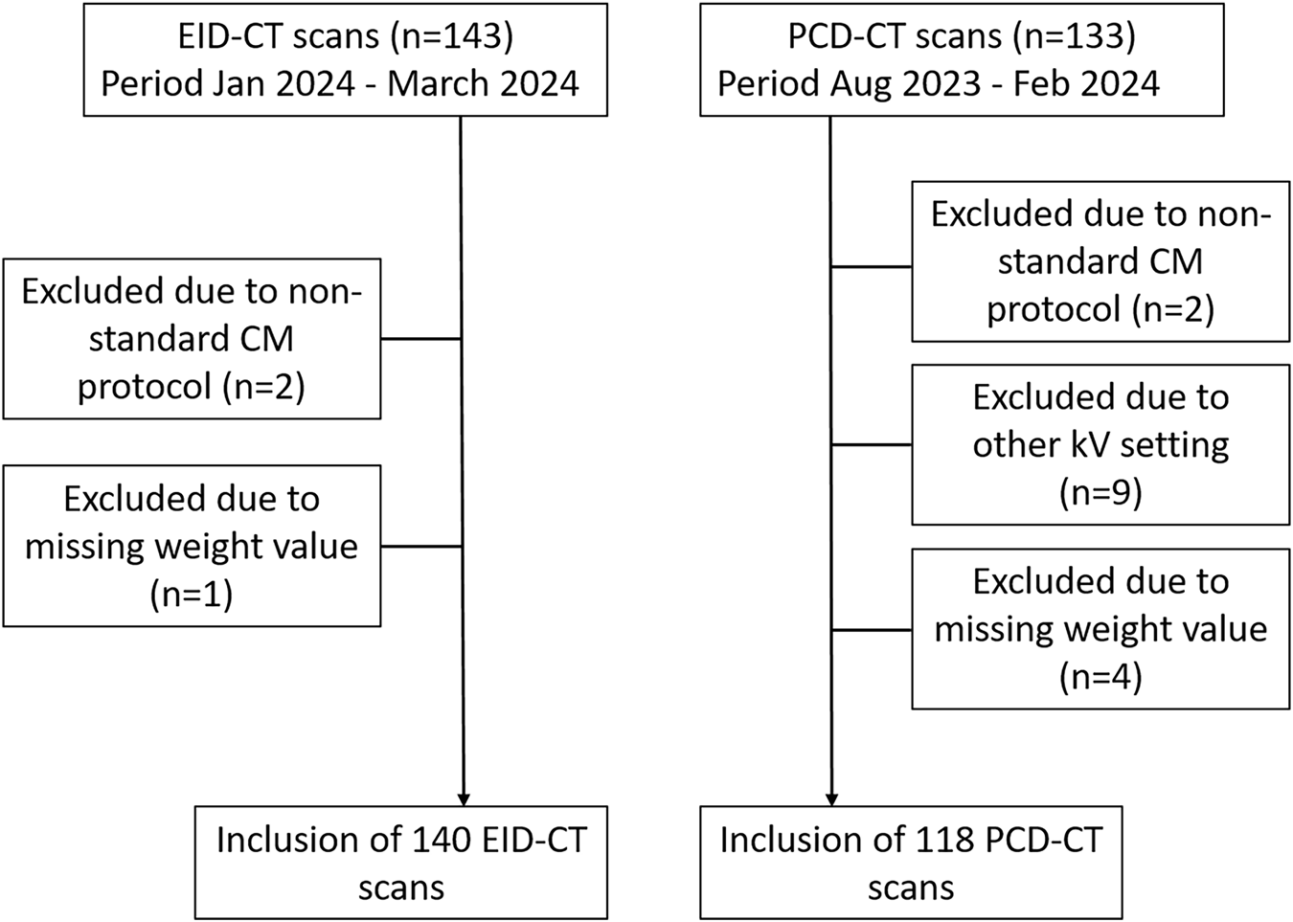
Flowchart showing patient selection process. EID, energy-integrating detector; PCD, photon-counting detector; CM, contrast media

A total of 143 EID-CT CTPAs and 133 PCD-CT CTPAs were performed during the inclusion periods. After exclusions, the final dataset comprised 140 EID-CT (mean age, 64.3 ± 15.6, 66 women) and 118 PCD-CT scans (mean age, 64.3 ± 15.3, 72 women) (Fig. 1). The majority of exclusions for the PCD-CT were due to the use of a tube voltage of 140 kV rather than the specified 120 kV (*n* = 9). Characteristics of the study population per CT scanner are summarized in Table 1 (and Appendix Table 1). A significant difference in sex distribution (*p* = 0.03) was demonstrated between the two groups, while other characteristics showed no statistically significant differences.

**Table 1:** Baseline characteristics

Parameters	EID-CT scans (*n* = 140)	PCD-CT scans (*n* = 118)	*p*
Age [years]	64.3 ± 15.6	64.3 ± 15.3	0.90*
Gender [% female]	47.1%	61.0%	0.03†
TBW [kg]	80.1 ± 18.8	78.3 ± 18.6	0.47^*^
Height [cm]	172 ± 10	170 ± 9	0.09°
BMI [kg/m^2^]	27.1 ± 5.5	26.7 ± 5.3	0.64*

*EID* energy-integrating detector, *PCD* photon-counting detector, *TBW* total body weight, *BMI* body mass index

*Mann–Whitney *U*-test

†Chi-square test

°Independent sample *T*-test

Table 2 summarizes CM and radiation dose parameters, showing that TIL, IDR, and DF were significantly lower for the PCD-CT. A 26.7% reduction in TIL was found in the PCD-CT group compared to the EID-CT group (10.2 ± 1.4 gI vs 13.9 ± 4.1 gI, respectively, *p* = 0.000). Furthermore, CTDI_vol_ was higher for the EID-CT group than for the PCD-CT group (4.5 ± 2.7 mGy vs 3.4 ± 1.1 mGy, with a reduction of 24.4%, *p* < 0.001).

**Table 2:** CM injection and radiation dose parameters

Parameters	EID-CT scans (*n* = 140)							PCD-CT scans (*n *= 118)	p*
	70 kV (*n* = 26)	80 kV (*n* = 38)	90 kV (*n* = 40)	100 kV (*n* = 23)	110 kV (*n* = 6)	120 kV (*n* = 7)	All (*n* = 140)		
TIL [gI]	9.6 ± 2.3	11.9 ± 1.5	14.3 ± 2.1	17.6 ± 2.5	18.7 ± 3.2	22.9 ± 4.3	13.9 ± 4.1	10.2 ± 1.4	0.000
IDR [gI/s]	0.8 ± 0.2	1.0 ± 0.1	1.2 ± 0.2	1.5 ± 0.2	1.5 ± 0.2	1.9 ± 0.3	1.2 ± 0.4	0.8 ± 0.1	< 0.001
DF [gI/kg]	0.15 ± 0.03	0.16 ± 0.02	0.18 ± 0.02	0.19 ± 0.02	0.22 ± 0.03	0.22 ± 0.03	0.17 ± 0.03	0.13 ± 0.02	0.000
CTDI_vol_ [mGy]	1.8 ± 0.5	3.3 ± 0.9	4.7 ± 1.1	6.4 ± 1.3	7.2 ± 1.5	12.0 ± 3.7	4.5 ± 2.7	3.4 ± 1.1	< 0.001

*EID* energy-integrating detector, *PCD* photon-counting detector, *TIL* total iodine load, *IDR* iodine delivery rate, *DF* dosing factor, *CTDIvol* CT dose index volume

^*^Mann–Whitney *U*-test

### Objective IQ

Detailed results of the objective IQ assessment can be found in Table 3. For the primary outcome, both proximal and distal mean attenuation of the pulmonary arteries were not significantly different between the two groups (*p* = 0.66 and *p* = 0.71, respectively). Results for the secondary outcomes showed comparable SNR values between both groups, in both proximal and distal segments (*p* = 0.32 and *p* = 0.13, respectively). CNR was slightly higher for the proximal pulmonary arteries at EID-CT (18.7 ± 9.3) vs PCD-CT (15.9 ± 5.9; *p* = 0.02), whereas the CNR for the distal pulmonary arteries did not demonstrate a significant difference (17.8 ± 9.2 and 15.6 ± 5.9 for the EID-CT and PCD-CT, respectively, *p* = 0.09). Stratified analyses were conducted to account for the significant gender difference between the two groups (see Appendix Table 2). Results showed no significant differences except for the CNR among females in both the proximal and distal pulmonary arteries (*p* = 0.003 and *p* = 0.02, respectively), as well as in SNR within the distal pulmonary arteries (*p* = 0.01).

**Table 3:** Objective IQ outcomes

	EID-CT scans (*n* = 140)	PCD-CT scans (*n* = 118)	p*
Proximal			
Mean attenuation (± SD) [HU]	384.7 ± 105.8	378.9 ± 100.6	0.66
SNR (± SD)	14.9 ± 4.2	14.4 ± 4.1	0.32
CNR (± SD)	18.7 ± 9.3	15.9 ± 5.9	0.02
Distal			
Mean attenuation (± SD) [HU]	368.7 ± 107.4	373.6 ± 101.4	0.71
SNR (± SD)	20.5 ± 6.7	19.2 ± 5.2	0.13
CNR (± SD)	17.8 ± 9.2	15.6 ± 5.9	0.09

*EID* energy-integrating detector, *PCD* photon-counting detector, *HU* Hounsfield unit, *SD* standard deviation, *SNR* signal-to-noise ratio, *CNR* contrast-to-noise ratio

*Mann–Whitney *U*-test

### Subjective IQ

**Fig. 2:**
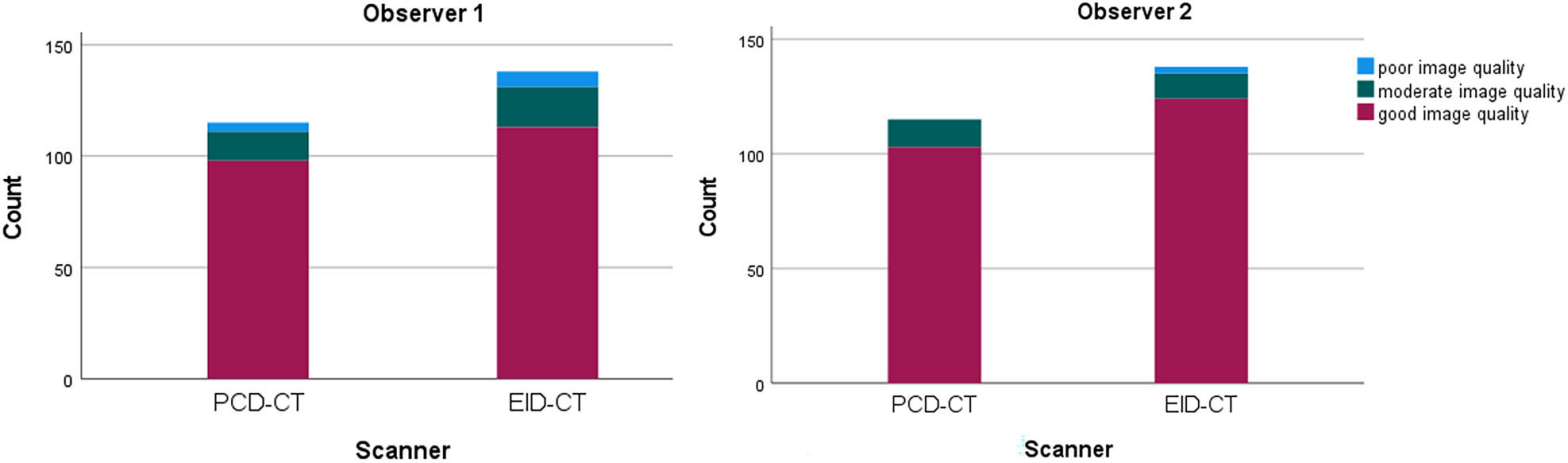
Results of subjective IQ assessment by 2 observers. PCD, photon-counting detector; EID, energy-integrating detector

**Fig. 3:**
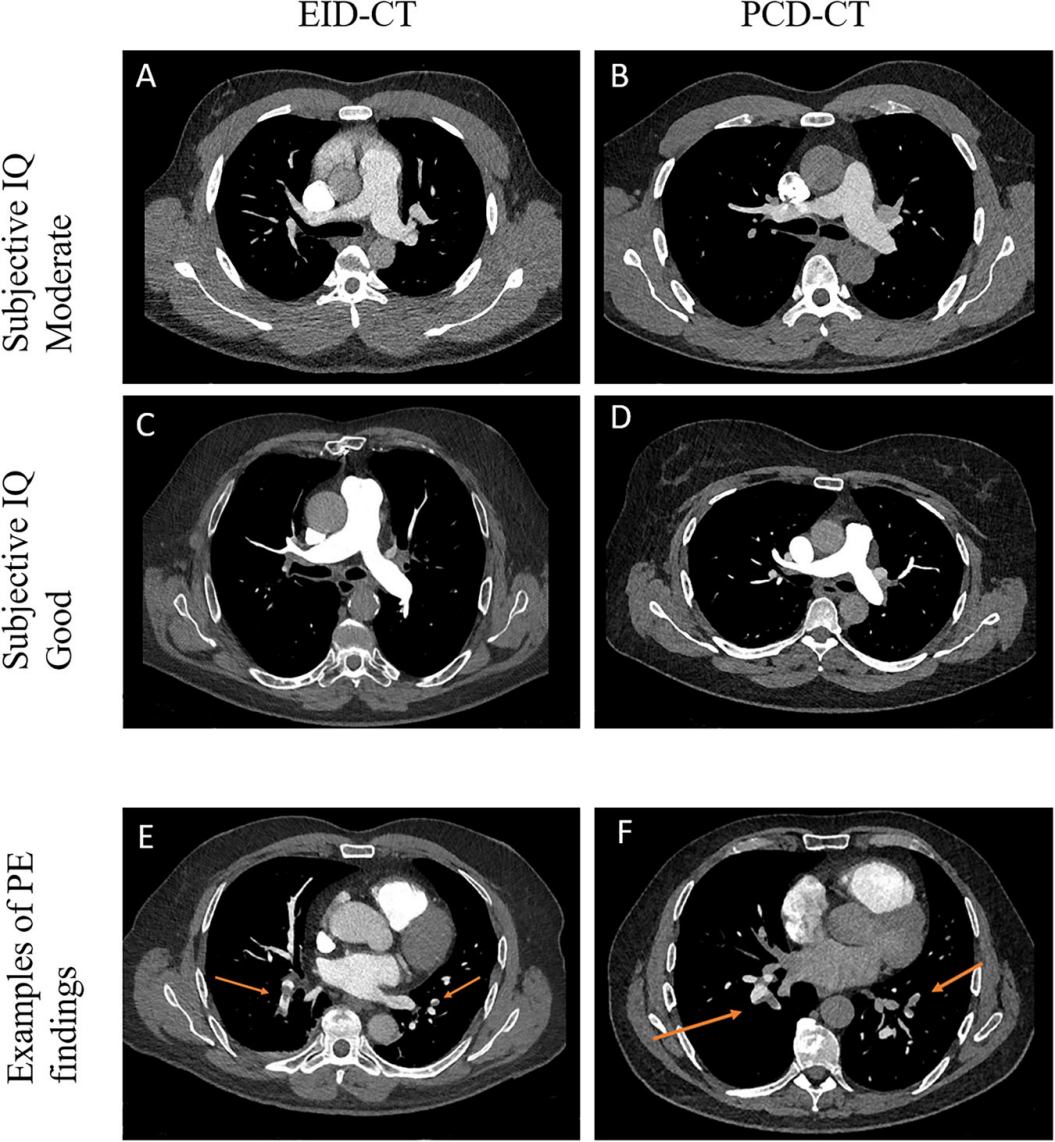
Representative CT pulmonary artery scans from both a PCD-CT and an EID-CT. Images **A**, **B** were rated by both observers as moderate, and **C**, **D** were rated as good by both observers. Images **E**, **F** are examples of PE findings for both scanners

Subjective IQ assessment showed a rating of moderate or good IQ in 94.9% of the EID-CT scans and 96.5% of the PCD-CT scans for observer 1. Observer 2 rated 97.7% of EID-CT and 100% of PCD-CT scans as having moderate or good IQ (Fig. 2). There was a good interobserver agreement, with an ICC of 0.667 (95% CI: 0.574–0.740). Representative images are shown in Fig. 3 and illustrate comparable IQ, including an example of PE findings on both scanners.

## Discussion

This study evaluated the potential for reducing CM in CTPA scans using PCD-CT compared to EID-CT, utilizing tailored CM protocols based on TBW for the PCD-CT and highly individualized CM protocols adapted for both tube voltage and TBW for the EID-CT. The results indicate that a substantial and additional CM reduction is achievable with PCD-CT in a clinical setting, while maintaining IQ and decreasing radiation dose compared to an individualized and optimized EID-CT protocol on a 3rd-generation dual-source CT. While the TBW limits the use of low kV with correspondingly increased iodine contrast in EID-CT because of limited X-ray tube power reserves, patients can be examined on the PCD-CT with the standard protocol at 120 kV regardless of their body weight, and the reconstruction of VMIs at 55 keV is also always possible. Specifically, using PCD-CT resulted in a 26.7% reduction in TIL and a 24.4% reduction in CTDI_vol_. Although the potential reduction in patient risk from lowering CM dosage may be subject to debate, this approach offers environmental and financial benefits, while minimizing radiation dose remains a consistent priority.

Objective IQ analysis showed no significant differences in attenuation, SNR, and CNR values between PCD-CT and EID-CT except for CNR measured in the proximal pulmonary arteries, which exhibited a significantly lower CNR on the PCD-CT compared to EID-CT. However, this lower CNR is not considered clinically relevant, as it is likely due to increased noise in the paraspinal muscle, which served as the reference region for noise calculation but is not of primary interest in the study. This increase in noise may be attributed to cross-scatter correction effects, caused by dual source scanning used for the PCD-CT [37]. Therefore, in future studies, it may be advised to use an alternative reference region in CNR calculations, e.g., the cardiac septum [35]. Furthermore, subjective IQ analysis showed that almost all CT scans, and more PCD-CT than EID-CT scans, were rated as having moderate or good IQ, indicating sufficient diagnostic IQ.

Previous studies have demonstrated that using a PCD-CT for CTPA can enable a 20–50% CM reduction compared to EID-CT, while still maintaining diagnostic IQ, which is consistent with the findings of the present study [25, 27]. However, these studies employed fixed CM protocols not adjusted for TBW and, for the EID-CT, not adjusted for tube voltage or TBW. Another difference is the comparison of spectral PCD-CT with SE EID-CT in the present study, as opposed to the DE EID-CT mode utilized in the previous study [25]. With EID-CT, both SE and DE CTPA are used in daily clinical practice. Vlahos et al (2022), and Yadav et al (2023), demonstrated that DE CTPA offers superior diagnostic sensitivity compared to SE CTPA [38, 39]. In addition, it may provide additional diagnostic capabilities by providing deeper physiological insights and hemodynamic correlates, which could inform and optimize the management of patients with pulmonary hypertension [38]. On the other hand, in a study by Chen et al (2019), DE CTPA did not outperform SE CTPA with regard to diagnostic IQ [40], and previous studies demonstrated a lower radiation dose and significantly reduced scan time for SE CTPA compared to DE CTPA, the latter being particularly advantageous for patients experiencing shortness of breath [26]. Additionally, limitations regarding DE CTPA on EID-CT were described, depending on the DE configuration of the scanner (e.g., limited field of view and slight temporal misregistration) [38].

These limitations are overcome by PCD-CT for PE assessment. The availability of spectral data enables reconstruction of VMIs at lower keV levels for scans with insufficient contrast attenuation. This increases the iodine contrast in the image, potentially reducing the need for repeat scans and thus avoiding increased CM and radiation doses [22, 41]. However, further research is required to investigate this potential improvement.

This study has several limitations. First, it is a retrospective single-center study with a relatively small sample size. A prospective study design would have allowed for adjustments to improve consistency between the two scanners, although differences in settings will always exist due to the differences in scanner technique between EID-CT and PCD-CT. However, the retrospective study design provided the opportunity to examine their performance in routine daily clinical practice. Second, the use of Likert scores is inherently subjective, as observers tend to avoid selecting extreme scores and may interpret the scale differently [42]. To mitigate this issue, we grouped the lowest scores and the highest scores into categories of poor and good IQ, respectively. Moreover, since the primary aim of the subjective IQ assessment is to determine whether both scanners provide sufficient diagnostic IQ (moderate/good), small variations in the specific Likert scores between observers (e.g., due to difference in observer experience, or due to familiarity to specific scanner types) are not of major concern, even though they affect the ICC for subjective IQ assessment. As shown in Fig. 2, both observers classified the majority of scans as having sufficient diagnostic IQ, which aligns with the study’s primary objective. A third limitation was the lower pitch of 1.3 used for the EID-CT in our study, resulting from a standardization of EID-CT scan protocols in our institution. The minimal difference in scan time (± 1 s) and the consistent delay used for both scanners, suggest this pitch variation does not impact contrast enhancement. Lastly, a difference in the percentage of female and male participants was observed between the EID-CT and PCD-CT (47.1% vs 61.0%, respectively). Females generally have a lower blood volume compared to males [43]. A lower blood volume might lead to increased iodine concentration in the blood and, as a result, increased attenuation [44]. Stratified analysis showed only significant differences in CNR in females within both the distal and proximal pulmonary arteries, along with a significant difference in SNR in the distal pulmonary arteries. This indicates that the unequal gender distribution between the two groups, may have introduced bias into these results. However, the mean outcome measures did not show clinically relevant differences. More importantly, attenuation, the primary outcome of this study, showed no significant differences in analyses stratified by gender. Nevertheless, evidence for sex-based differences in attenuation remains limited, and further studies are needed to examine this phenomenon [44].

In conclusion, this study compared TBW-adapted CM protocols for CTPA scans on a PCD-CT with highly individualized kV- and TBW-adapted protocols on EID-CT and demonstrated that PCD-CT can achieve substantial reductions in CM and radiation dose, while maintaining sufficient diagnostic IQ.

## Supplementary information

Supplementary information

## Abbreviations

CM: Contrast media
CNR: Contrast-to-noise ratio
CTPA: CT pulmonary angiography
EID-CT: Energy-integrating detector CT
IQ: Image quality
PCD-CT: Photon-counting detector CT
PE: Pulmonary embolism
SNR: Signal-to-noise ratio
TBW: Total body weight
VMI: Virtual mono-energetic images
