# Supplementary material for: Reducing contrast media dosage for pulmonary embolism CTPA in PCD-CT: a comparative study of EID-CT and PCD-CT in the era of individualized protocolling
Source: Eur Radiol. 2025 Oct 17;36(4):3206–15. doi: 10.1007/s00330-025-12054-6 (PMC13035631; doi:10.1007/s00330-025-12054-6)
Supplement: Supplementary file 1 — Supplementary information [file 330_2025_12054_MOESM1_ESM.pdf]

Appendix

TABLE A1. Baseline characteristics

Parameters	***EID-CT scans (n = 140)***							***PCD-CT scans (n = 118)***	***p****
	70 kV (n = 26)	80 kV (n = 38)	90 kV (n = 40)	100 kV (n = 23)	110 kV (n = 6)	120 kV (n = 7)	All (n = 140)		
Age [years]	64.1 ± 18.3	62.5 ± 16.9	67.1 ± 14.3	64.8 ± 12.9	70.0 ± 11.1	52.9 ± 12.1	64.3 ± 15.6	64.3 ± 15.3	0.90*
Gender [% female]	80.8%	39.5%	50.0%	26.1%	33.3%	28.6%	47.1%	61.0%	0.03†
TBW [kg]	62.7 ± 13.1	75.4 ± 11.5	82.1 ± 13.4	94.7 ± 18.9	85.3 ± 3.6	106.6 ± 31.5	80.1 ± 18.8	78.3 ± 18.6	0.47*
Height [cm]	166 ± 7.5	172 ± 9	171 ± 10	176 ± 9	174 ± 7	179 ± 12	172 ± 10	170 ± 9	0.09°
BMI [kg/m^2]	22.8 ± 4.6	25.6 ± 3.3	28.1 ± 4.3	30.6 ± 6.8	28.2 ± 2.4	32.9 ± 8.2	27.1 ± 5.5	26.7 ± 5.3	0.64*

*Mann-Whitney U test; † Chi-square test; °Independent sample T-test

EID, energy integrating detector; PCD, photon counting detector; TBW, total body weight; BMI, body mass index.

TABLE A2. Objective image quality outcomes stratified by Gender

	EID-CT scans (Male, n = 74 Female, n = 66)	*PCD-CT scans (Male, n = 46 Female, n = 72)*	*p**
Proximal			
Mean attenuation (±SD) [HU]			
Male	368.7 ± 125.9	376.1 ± 111.3	0.54
Female	402.7 ± 74.5	380.6 ± 93.9	0.11
SNR (±SD)			
Male	14.1 ± 4.7	13.7 ± 3.4	0.94
Female	15.8 ± 3.3	14.9 ± 4.5	0.06
CNR (±SD)			
Male	17.8 ± 10.7	15.0 ± 5.1	0.45
Female	19.7 ± 7.3	16.5 ± 6.4	0.003
Distal			
Mean attenuation (±SD) [HU]			
Male	349.4 ± 122.1	365.4 ± 115.4	0.36
Female	390.4 ± 83.7	378.8 ± 91.9	0.41
SNR (±SD)			
Male	19.5 ± 7.3	19.0 ± 5.1	0.98
Female	21.5 ± 5.9	19.3 ± 5.2	0.01
CNR (±SD)			
Male	16.8 ± 10.6	14.4 ± 4.9	0.63
Female	19.0 ± 7.3	16.4 ± 6.4	0.02

*Mann-Whitney U test

EID, energy integrating detector; PCD, photon counting detector; HU, Hounsfield unit; SD, standard deviation; SNR, signal-to-noise ratio; CNR, contrast-to-noise ratio.
